# A short review on SSF – an interesting process option for ethanol production from lignocellulosic feedstocks

**DOI:** 10.1186/1754-6834-1-7

**Published:** 2008-05-01

**Authors:** Kim Olofsson, Magnus Bertilsson, Gunnar Lidén

**Affiliations:** 1 Department of Chemical Engineering, Lund University, Box 124, 221 00 Lund, Sweden

## Abstract

Simultaneous saccharification and fermentation (SSF) is one process option for production of ethanol from lignocellulose. The principal benefits of performing the enzymatic hydrolysis together with the fermentation, instead of in a separate step after the hydrolysis, are the reduced end-product inhibition of the enzymatic hydrolysis, and the reduced investment costs. The principal drawbacks, on the other hand, are the need to find favorable conditions (*e.g*. temperature and pH) for both the enzymatic hydrolysis and the fermentation and the difficulty to recycle the fermenting organism and the enzymes. To satisfy the first requirement, the temperature is normally kept below 37°C, whereas the difficulty to recycle the yeast makes it beneficial to operate with a low yeast concentration and at a high solid loading. In this review, we make a brief overview of recent experimental work and development of SSF using lignocellulosic feedstocks. Significant progress has been made with respect to increasing the substrate loading, decreasing the yeast concentration and co-fermentation of both hexoses and pentoses during SSF. Presently, an SSF process for *e.g*. wheat straw hydrolyzate can be expected to give final ethanol concentrations close to 40 g L^-1 ^with a yield based on total hexoses and pentoses higher than 70%.

## Introduction

Bioethanol produced by fermentation of lignocellulosic biomass (second generation bioethanol), from agricultural by-products, forest residues or energy crops, shows many potential advantages in comparison to sugar or starch-derived bioethanol (first generation bioethanol), from both energetic and environmental points of view. One significant environmental factor is that the reduction in greenhouse gas emission will be larger with lignocellulosic ethanol than for starch-derived ethanol, due to the lower overall oil input required in the process [1]. Most process concepts for bioethanol from lignocellulose start with a thermo-chemical hydrolysis of the hemicellulose part (pretreatment), followed by an enzymatic hydrolysis of the cellulose part and a yeast-based fermentation of the resulting sugars. Lignin, the main by-product in the process, can be directly used as solid fuel, or as a source for higher added-value biorefinery products. Highly encouraging progress has been made with respect to decreasing the cost of enzymes, optimizing the method of pretreatment, and developing novel yeast strains, primarily *Saccharomyces cerevisiae *strains capable of fermenting pentoses.

One option is to perform the enzymatic hydrolysis together with the fermentation, instead of subsequent to the enzymatic hydrolysis. This is called SSF – after *Simultaneous Saccharification and Fermentation*. SSF is today important in the dry-milling process in the corn-based ethanol industry in the U.S. [2]. In the current review, we look at recent developments on SSF applied to *lignocellulosic feedstocks*.

## The Process – step-by-step

### The SSF concept

The idea of performing the enzymatic hydrolysis and fermentation simultaneously was put forward by Gauss *et al*. in a patent from 1976 [3]. The authors stated that the glucose yield in a traditional separate enzymatic hydrolysis (using enzymes produced by the fungus *Trichoderma reesei*) was low, probably due to end-product inhibition of the hydrolysis by glucose and cellobiose. The authors could, however, show that they obtained a higher overall ethanol yield when using SSF, which they attributed to the removal of glucose and cellobiose by the fermentation, and the consequent release of end-product inhibition. The term SSF (the abbreviation SSF is often used also for *solid state fermentation*) was not used by the authors at the time, but became the common notation for this process within just a few years from the original invention. The avoidance of end-product inhibition is still probably the most important reason for using SSF, but there are several additional potential advantages. Gauss and co-workers, mentioned for instance the advantage that glucose does not need to be separated from the lignin fraction following a separate enzymatic hydrolysis step, thereby avoiding a potential loss of sugar. Furthermore, the combination of hydrolysis and fermentation decreases the number of vessels needed and thereby investment costs. The decrease in capital investment has been estimated to be larger than 20%. This is quite important, since the capital costs can be expected to be comparable to the raw material costs in ethanol production from lignocellulose [4]. Other advantages, relating to co-consumption of pentose and hexose sugars, and detoxification have become apparent more recently, as will be discussed later in this review.

**Figure 1:**
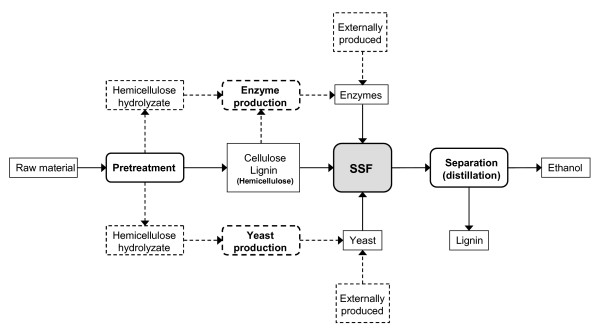
Schematic representation of an SSF process.

Inevitably, there are also disadvantages of SSF in comparison to the separate hydrolysis and fermentation (SHF) process. The optimum temperature for enzymatic hydrolysis is typically higher than that of fermentation – at least when using yeast as the fermenting organism. In an SHF process, the temperature for the enzymatic hydrolysis can be optimized independently from the fermentation temperature, whereas a compromise must be found in an SSF process. Furthermore, the yeast cannot be reused in an SSF process due to the problems of separating the yeast from the lignin after fermentation. Therefore, the yeast will necessarily represent a yield loss in an SSF process, if the yeast is produced from carbohydrates within the process (see Figure 1) or a running cost if it is externally supplied. The enzymes are equally difficult to reuse, also in an SHF process. The enzymes are either produced within the process (see Figure 1) – thereby representing a loss of substrate – or are externally supplied and thereby add to the chemical costs. Recirculation of enzymes is equally difficult since the enzymes bind to the substrate, although a partial desorption can be obtained after addition of surfactants [5].

The availability of lignocellulosic feedstocks varies depending on geographic location (see *e.g*. Kim and Dale [6]), and the lignocellulosic feedstocks are rather heterogeneous in terms of both structure and chemical composition (see Table 1). This heterogeneity has a strong impact on the process design, affecting virtually all process steps, *i.e*. the mechanical handling of the material, pretreatment conditions, choice of enzymes and yeast strains, as well as separation and properties of the remaining lignin. This will become apparent in the discussion below.

**Table 1:** Composition of some lignocellulosic raw materials (% of dry matter)

**Raw material**	**Glucan**	**Mannan**	**Galactan**	**Xylan**	**Arabinan**	**Lignin**	**Ref**
*Agricultural residues*							
Corn stover	36.4	0.6	1.0	18.0	3.0	16.6	[151]
Rice straw	34.2	-	-	24.5	-	11.9	[151]
Sugar cane bagasse	40.2	0.5	1.4	22.5	2.0	25.2	[152]
Wheat straw	38.2	0.3	0.7	21.2	2.5	23.4	[151]
Switch grass	31.0	0.3	0.9	20.4	2.8	17.6	[151]
*Hardwood*							
Salix	41.5	3.0	2.1	15.0	1.8	25.2	[153]
*Softwood*							
Pine	46.4	11.7	-	8.8	2.4	29.4	[151]
Spruce	49.9	12.3	2.3	5.3	1.7	28.7	[35]

### Pretreatment

The purpose of the pretreatment is to alter the lignocellulosic structure and increase the rate of enzymatic hydrolysis of primarily the cellulose. This should be done with a minimum formation of compounds, which inhibit the fermenting microorganisms [7]. The accessible surface area is regarded as one of the most important factors affecting the effectiveness of enzymatic cellulose degradation [8-12]. In native wood only a small fraction of the cell wall capillaries are accessible to the enzymes [13]. Pretreatment, however, increases the available area in several ways [12,14-16]; i) fragments and cracks are formed yielding increased area [14], ii) the hemicellulose fraction is hydrolysed which diminishes shielding effects [17,18], iii) the lignin also undergoes structural changes [10,14,19,20] and the wood is delignified to various degrees, depending on the pretreatment technology [21]. Thus, the shielding of microfibrils and occluding of pores, caused by lignin, can be removed. Other factors, believed to influence the digestibility in SSF, are the substrate crystallinity [11,22,23] and the degree of polymerization (DP) [24].

The pretreatment methods can be divided into physical and chemical methods, and combinations of these two are commonly used (see *e.g*. the review written by Mosier *et al*. [21]). The type of feedstock strongly affects the choice of pretreatment method. The hemicellulose is, for instance, acetylated to a high degree in xylan-rich materials. Since acetate is liberated during hydrolysis, the pretreatment of these materials is to some extent autocatalytic and require less added acid and milder process conditions. However, the liberated acetate adds to the toxicity of the hemicellulose hydrolyzates.

Ammonia fiber/freeze explosion (AFEX) pretreatment is regarded as an attractive method for pretreatment of agricultural residues, yielding highly digestible cellulose [25,26]. AFEX depolymerizes the lignin, removes the hemicellulose and decrystallizes the cellulose [27,28]. The moderate temperature and pH also minimize formation of sugar degradation products. However, the method suffers from high costs of ammonia and ammonia recovery [25]. In this context the lime method, based on calcium (or sodium) hydroxide [29-31] should also be mentioned. Alkali pretreatments are run at lower temperatures for long residence times, and as for the AFEX method, a delignification of the biomass is obtained.

Steam explosion is an intensively studied pretreatment method [21]. The effects of uncatalyzed steam explosion – and liquid hot water pretreatments – on the biomass are primarily attributed to the removal of hemicelluloses. By adding an acid catalyst, the hydrolysis can be further improved [19,32]. Dilute acid pretreatments using H_2_SO_4 _[33-36] or SO_2 _[37-41] are the most investigated pretreatment methods because of their effectiveness and inexpensiveness. These methods have been applied in pilot plants and, hence, are close to commercialization [42,43]. Acid catalyzed treatment improves the hemicellulose removal [19,32], gives a partial hydrolysis of cellulose [34,37,38] and alters the lignin structure [10,14,19,20]. The main drawbacks are related to the process equipment requirements [21,44] and inhibitor formation [45]. So far, successful pretreatments with alkali, AFEX and liquid hot water have been limited to agricultural residues and herbaceous crops [25,46-48], whereas acid catalysed steam pretreatments have generated high sugar yields from these materials as well as from softwood feedstocks [33-41].

A simple quantification of the harshness of a steam pretreatment process is the so called Severity Factor, *log*(*R*_0_). This factor combines the time and the temperature of a process into a single entity, $R_{0}=t\cdot e^{\frac{T_{r}-100}{14.75}}$[49]. For acid catalyzed pretreatments the Combined Severity Factor, log(*CS*), is sometime used. This takes also the pH into account, log(*CS*) = log(*R*_0_) - *pH*[50], and typical values for acid catalyzed steam explosion pretreatment of softwood are in the range 2 to 4 [35,41].

Optimal pretreatment conditions in an SSF process do not necessarily differ much from those of an SHF processes utilizing lignocellulosic biomass. However, several compounds present in pretreatment hydrolyzates, which inhibit enzymatic hydrolysis are converted by the fermenting organisms. This is a probable explanation behind the higher reported ethanol yields in SSF compared to SHF [51,52]. Inhibitor formation from the pretreatment may therefore be tolerated to a higher extent in an SSF process. Inhibitory compounds can be put into three major groups; furaldehydes, weak acids, and phenolics. The two most common furaldehydes, HMF (5-hydroxymethyl-2-furaldehyde) and furfural (2-furaldehyde), are formed at severe conditions from hexoses and pentoses, respectively [45,53,54]. Weak acids from lignocellulosic materials, such as acetic, formic and levulinic acid, are mainly formed by de-acetylation of hemicellulose or HMF breakdown [53,54]. Phenolic compounds are formed chiefly during lignin breakdown, and are to be found in numerous variants, depending on the type of lignin [55]. For a more in-depth discussion on inhibition see *e.g*. the review by Almeida et al [7].

### Enzymatic hydrolysis

A successful pretreatment has to a large extent removed the hemicellulose, leaving the cellulose available for hydrolysis. Since the most commonly used microorganisms for ethanol production solely utilize sugar monomers, the cellulose needs to be hydrolyzed, which in an SSF occurs concomitantly with the fermentation. Historically, industrial cellulose digestion has been made with acid hydrolysis [56] and optimization of acid hydrolysis of various lignocellulosic materials have been carried out for ethanol producing purposes [57-59]. Acid hydrolysis, however, produces hydrolyzates that are relatively toxic to the fermenting microorganisms, and the maximum glucose yield is limited to approximately 60% in a batch process for kinetic reasons [60]. Enzymatic degradation of the cellulose fraction, on the other hand, has the potential of yielding relatively non-toxic hydrolyzates with higher sugar yields.

Enzymes specialized in breaking up the β-1-4-glycosidic bonds of glucan are collectively called cellulases. In 1950, Reese et al [61] presented a model of enzymatic cellulose hydrolysis based on multiple enzymes (C_1 _and C_X_). The C_1 _enzyme was assumed to produce shorter polyanhydro-glucose chains, while the solubilization was attributed to the C_X _enzyme. Basically the same picture applies today, but there has been a huge progress in knowledge about all the different specific enzyme components involved. The cellulases are divided into three sub-categories, representing three types of activity: endoglucanases, exoglucanases (cellobiohydrolases) and β-glucosidases. Endoglucanases significantly reduce the degree of polymerization of the substrate by randomly attacking the interior parts, mainly in the amorphous regions of cellulose. Exoglucanases (or cellobiohydrolases), on the other hand, incrementally shorten the glucan molecules by binding to the glucan ends and releasing mainly cellobiose units. Finally, β-glucosidases split the disaccharide cellobiose into two units of glucose.

Several types of microorganisms can produce cellulase systems including aerobic filamentous fungi, aerobic actinomycetes, anaerobic hyperthermophilic bacteria and anaerobic fungi (see *e.g*. review by Lynd *et al*. [62]). Intensive research on the aerobic filamentous fungi *T. reesei *during the past decades has resulted in an efficient cellulase producing organism, which is currently dominating the industrial cellulase production [62,63].

As already mentioned, an important advantage with SSF compared to SHF is the reduction of end-product inhibition by sugars formed in the hydrolysis. The fermentation product ethanol also inhibits hydrolysis, but to a lesser extent than cellobiose or glucose [64]. Another advantage is that inhibitors from the pretreatment can be metabolized by the microorganisms [51]. However, also the SSF process may suffer from incomplete hydrolysis of the solid lignocellulosic fraction. Except for inhibition by end-products or other components [51,65], this can be due to enzyme deactivation, unproductive enzyme adsorption [66], decreasing availability of chain ends [24], and increasing crystallinity with conversion of pretreated cellulose [67].

In an industrial SSF, enzyme and cell concentrations should be appropriately balanced in order to minimize costs for yeast and enzyme production. Synergies between the enzymes, *e.g*. endo-exo synergism [68,69], exo-exo synergism [70], and synergism between endo- or exoglucanases and β-glucosidases [71], should also be optimized by tuning the composition of the enzyme mixtures. The optimal composition will most certainly depend on the lignocellulosic raw material.

### Fermenting microorganisms

The general requirements on an organism to be used in ethanol production is that it should give a high ethanol yield, a high productivity and be able to withstand high ethanol concentrations in order to keep distillation costs low [72]. In addition to these general requirements, inhibitor tolerance, temperature tolerance and the ability to utilize multiple sugars are essential for SSF applications. Tolerance towards low pH-values will minimize the risk of contamination. The work-horse in starch or sucrose-based ethanol production is the common Bakers' yeast, *Saccharomyces cerevisiae*. This organism produces ethanol at a high yield (higher than 0.45 g g^-1 ^at optimal conditions) and a high specific rate (up to 1.3 g g^-1 ^cell mass h^-1 ^[73]). It also has a very high ethanol tolerance, over 100 g L^-1 ^has been reported for some strains and media [74]. In addition, the organism has proven to be robust to other inhibitors, and hence it is suitable for fermentation of lignocellulosic materials [75,76].

Hemicellulose from hardwood and agricultural residues are typically rich in xylans (cf. Table 1) – hardwood containing primarily O-acetyl-4-O-methyl-glucuronoxylan, whereas grasses contain arabinoxylan [77]. Softwood hemicellulose, on the other hand, contains more mannans – primarily in the form on galactoglucomannan – and less xylan. Mannose fermentation is normally efficient in *S. cerevisiae*, whereas the ability to ferment galactose is strain dependent [78], and the genes for galactose utilization are furthermore repressed by glucose [79,80], leading to a typical sequential utilization of the sugars. Clearly, xylose fermentation is a more significant issue for agricultural residues and hardwood than for softwood. Xylose is not metabolized by wild-type *S. cerevisiae*, apart from a minor reduction to xylitol. This, and for some parts the temperature tolerance, have been the main reason behind the interest to test also other microorganisms for lignocellulose conversion in SSF.

Naturally xylose-fermenting yeasts, such as *Pichia stipitis *and *Candida shehatae *[81-83], could potentially be advantageous to use in SSF of materials with high xylan contents. However, their tolerance to inhibitory compounds in undetoxified lignocellulose hydrolyzates is rather low [84,85], and in addition, a very low and well-controlled supply of oxygen is required for efficient xylose fermentation [86-88]. The main "competitors" to the yeast have been the bacteria *Zymomonas mobilis *and genetically engineered *Escherichia coli*. *Z. mobilis*, an obligately anaerobic bacterium, which lacks a functional system for oxidative phosphorylation, produces ethanol and carbon dioxide as principal fermentation products. Interestingly, *Z. mobilis *utilizes the Entner-Duodoroff pathway which gives a lower ATP production per catabolized glucose [89,90]. This in turn gives a lower biomass yield and a higher ethanol yield on glucose compared to *S. cerevisiae *[91]. However, wild-type *Z. mobilis *lacks the ability to ferment pentose sugars, and a major drawback is furthermore that it is not a very robust organism. In general, bacteria appear to be less tolerant to lignocellulose-derived inhibitors [92], and a detoxification step may be needed prior to the fermentation. In contrast to Bakers' yeast and *Z. mobilis*, *E. coli *is capable of metabolizing a wide variety of substrates (including hexoses, pentoses and lactose), but the wild-type organism has a mixed fermentative pathway, and is thus a poor ethanol producer. In a landmark contribution, awarded U.S. patent number 5000000 [93], a strain of *E. coli *was genetically engineered into an ethanol producer by overexpression of *PDC *(encoding pyruvate decarboxylase) and *adhB *(encoding alcohol dehydrogenase) from *Z. mobilis *[94]. Excellent results have been achieved with recombinant *E. coli*, *e.g*. the KO11 strain, which have shown ethanol yields from 86 to close to 100% of the theoretical, and final ethanol concentrations up to 40 g L^-1 ^on hemicellulose hydrolyzates of bagasse, corn stover and corn hulls [95]. However, only the liquid fraction was used in reported studies, and the hydrolyzates were furthermore detoxified prior to use by overliming to pH 9 with calcium hydroxide and then adjusted to pH 6.0–6.5 with HCl. Furthermore, since the optimal pH is 6.5, *E. coli *is less suitable for SSF processes with *T. reesei *cellulases, which generally is considered to have a pH optimum around 4.8 [96].

### Pentose fermentation by engineered *S. cerevisiae*

Due to the very attractive properties of *S. cerevisiae *in industrial fermentations, there have been significant efforts made in the past decades to design recombinant xylose and arabinose fermenting strains of this yeast. Xylose fermenting strains of *S. cerevisiae *can in principal be constructed either by introducing genes encoding xylose isomerase (XI) from bacteria and fungi [97-99], or genes encoding xylose reductase (XR) and xylitol dehydrogenase (XDH) from fungi [100,101]. Also the endogenous *XKS1 *gene encoding xylulokinase (XK) has to be overexpressed to obtain significant xylose fermentation [101]. Transport proteins are needed for uptake of xylose, as well as of other sugars in yeast. In *S. cerevisiae*, xylose has been found to be transported by the hexose transporters, [102,103], but the affinity for xylose is approximately 200-fold lower than for glucose [104]. Consequently, xylose uptake is competitively inhibited by glucose.

**Figure 2:**
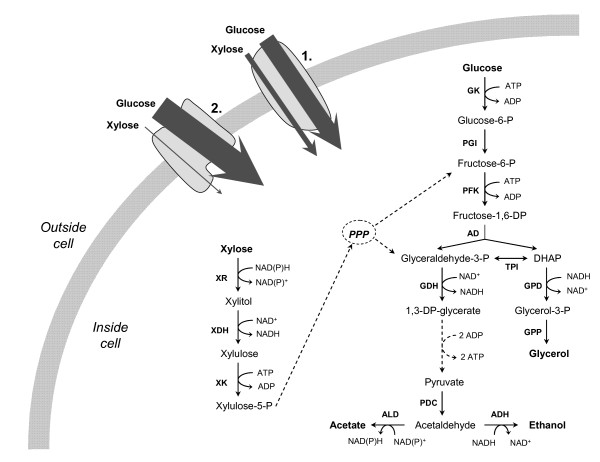
Simplified scheme of sugar transport and metabolism in *S. cerevisiae*. 1. Low- and intermediate-affinity hexose transporters. 2. High-affinity hexose transporters. (Abbreviations: PPP, pentose phosphate pathway; XR, xylose reductase; XDH, xylitol dehydrogenase; XK, xylulokinase; GK, glucokinase; PGI, phosphoglucose isomerase; PFK, phosphofructokinase; AD, aldolase; TPI, triose phosphate isomerase; GDH, glyceraldehyde-3-P dehydrogenase; GPD, glycerol-3-P dehydrogenase; GPP, glycerol-3-phosphatase; PDC, pyruvate decarboxylase; ALD, acetaldehyde dehydrogenase; ADH, alcohol dehydrogenase)

There are 20 different genes encoding sugar transport related proteins, 18 individual systems (Hxt1-17 and Gal2) and two related signal proteins (Snf3p and Rgt2p). The transporters exhibit different affinities for sugars, and the expression of their corresponding genes is regulated by the sugar concentrations, *i.e*. the availability of the carbon source [105]. It has previously been suggested that xylose is taken up by both high- and low-affinity systems of glucose transporters (Figure 2), but the uptake is increased in the presence of low glucose concentrations [106]. Studies have indicated that the high- and intermediate-affinity hexose transporters; Hxt4, Hxt5 Hxt7 and Gal2 are in fact the most important transporters for xylose [107]. Furthermore, it has been shown that a low (but non-zero) glucose concentration is needed in the medium for efficient xylose uptake [108]. This has been explained by a need for glucose for expression of glycolytic enzymes and intermediates [109], as well as generation of intermediary metabolites for the initial steps of the xylose metabolism and the pentose phosphate pathway [108]. Another possible explanation, inferred from both experiments and computer modeling, is that the glucose is needed for the expression of hexose transporters with favorable xylose transport properties, *e.g*. Hxt4 [110,111]. Consequently, in order to obtain efficient co-fermentation of xylose and glucose in SSF (sometimes denoted SSCF – simultaneous saccharification and co-fermentation) with recombinant *S. cerevisiae*, it is necessary to keep the glucose concentration low, which has been shown in practice in recent SSF studies [112,113].

## Experimental work on optimizing SSF

Reported experimental work on SSF have focused on improving the process by increasing the substrate loading (*i.e*. the content of water insoluble solids, WIS), decreasing enzyme and yeast concentration, and varying temperature and pH. Some recent SSF studies on lignocellulosic feedstocks, which have been made with reasonably high contents of water insoluble solids (WIS), and acceptable ethanol yields are shown in Tables 2 and 3. Many studies on pure cellulose (*e.g*. Sigmacell 50) have also been made, but these are not considered in this review. Table 2 shows studies in which only hexose sugars have been fermented, whereas Table 3 shows studies with co-fermentation of both hexoses and the pentose xylose.

**Table 2:** Brief summary of SSF experiments carried out on hexose sugars.

**Raw material**	**Type of pretreatment**	**Amount of solids ^1^**	**Detoxification**	**Temp (°C)**	**Strain**	**Cultivation on hydrolyzate**	**Final ethanol conc. (g L^-1^)**	**Ethanol yield (%) ^2^**	**Mode of operation ^3^**	**Year and reference**
Barley straw	Steam	7.5% WIS	No	35	*S. cerevisiae*	Yes	22.4	80	Batch	2007 [121]
Salix	Steam	9% WIS	No	37	*S. cerevisiae*	Yes	32	76	Batch	2006 [120]
Salix	Steam	11% WIS	No	37	*S. cerevisiae*	Yes	33	62	Fed-batch	2006 [120]
Spruce	Steam	10% WIS	No	37	S. *cerevisiae*	Yes	44.5	84	Batch	2005 [117]
Spruce	Steam	6–10% WIS	No	37	S. *cerevisiae*	Yes	44.5	84	Fed-batch	2005 [117]
Yellow Poplar Hardwood	Dilute-acid	11.5% WIS	LLE-OL ^4^	34	*Z. mobilis*	No	32.2	54	Batch	1999 [131]
Poplar	Steam	10% w/v	No (only solids in def. medium)	42	*K. fragilis *CECT 10875	No	19.0	71.2	Shake flask	2004 [138]
Eucalyptus	Steam	10% w/v	No (only solids in def. medium)	42	*K. fragilis *CECT 10875	No	17.0	62.5	Shake flask	2004 [138]
Wheat straw	Steam	10% w/v	No (only solids in def. medium)	42	*K. fragilis *CECT 10875	No	18.1	62.5	Shake flask	2004 [138]
Sweet sorghum bagasse	Steam	10% w/v	No (only solids in def. medium)	42	*K. fragilis *CECT 10875	No	16.2	60.9	Shake flask	2004 [138]
B. carinata residue	Steam	10% w/v	No (only solids in def. medium)	42	*K. fragilis *CECT 10875	No	19.0	68.1	Shake flask	2004 [138]
Old corrugated cardboard (OCC)	none	6 wt %	No, def. medium	40	*K. fragilis*	No	14.1	61.2 *	Shake flask	2004 [154]
Old corrugated cardboard (OCC)	none	6 wt %	No, def. medium	40	*S. cerevisiae*	No	14.2	61.8 *	Shake flask	2004 [154]
Paper sludge	none	6 wt %	No, def. medium	40	*K. fragilis*	No	8.8	63.7 *	Shake flask	2004 [154]
Paper sludge	none	6 wt %	No, def. medium	40	*S. cerevisiae*	No	9.0	65.5 *	Shake flask	2004 [154]
Antigonum leptopus leaves	Alkaline + H_2_O_2_	10% w/v	No	43	*K. fragilis*	No	27	n.a.	Shake flask	2001 [141]
Antigonum leptopus leaves	Alkaline + H_2_O_2_	10% w/v	No	40	*S. cerevisiae*	No	21	n.a.	Shake flask	2001 [141]
Sugar cane leaves	Alkaline + H_2_O_2_	10% w/v	No	43	*K. fragilis*	No	28	n.a	Shake flask	2001 [141]
Sugar cane leaves	Alkaline + H_2_O_2_	10% w/v	No	40	*S. cerevisiae*	No	22	n.a.	Shake flask	2001 [141]
Willow (Salix caprea QO82)	Steam- pretreatment	10% dry matter	No	37	*S. cerevisiae*	No	28.7	84.4	Batch	1995 [122]
Willow (Salix caprea QO82)	Steam	10% dry matter	No	37	*Z. mobilis*	No	27.9	82.1	Batch	1995 [122]
Switchgrass	Dilute sulfuric acid	7.5% w/v cellulose	No (only solids in def. medium)	37	*S. cerevisiae & B. clausenii *mixed culture	No	37.0 *	87 ^5^	Shake flask	1992 [136]
Sweetgum	Dilute sulfuric acid	7.5% w/v cellulose	No (only solids in def. medium)	37	*S. cerevisiae*	No	36.6 *	86 ^5^	Shake flask	1992 [136]
Corn cob	Dilute sulfuric acid	7.5% w/v cellulose	No (only solids in def. medium)	37	*S. cerevisiae*	No	39.1 *	94 ^5^	Shake flask	1992 [136]
Corn stover	Dilute sulfuric acid	7.5% w/v cellulose	No (only solids in def. medium)	37	*S. cerevisiae*	No	39.1 *	92 ^5^	Shake flask	1992 [136]
Wheat straw	Dilute sulfuric acid	7.5% w/v cellulose	No (only solids in def. medium)	37	*S. cerevisiae*	No	38.3 *	90 ^5^	Shake flask	1992 [136]
Populus	Dilute sulfuric acid	7.5% w/v cellulose	No (only solids in def. medium)	37	*S. cerevisiae*	No	38.3 *	90 ^5^	Shake flask	1991 [125]
Populus	Dilute sulfuric acid	7.5% w/v cellulose	No (only solids in def. medium)	37	*S. cerevisiae*	No	36.6 *	86 ^5^	Batch	1991 [125]

* Not directly given in the reference article, calculated by the authors.

1. The amount of solids can vary significantly due to how this is reported (*e.g*. WIS or dry matter), and may have a large impact on the SSF results. This is not always clearly defined in the respective research article.

2. Based on maximal theoretical ethanol yield on available hexoses, in most cases only glucose.

3. Batch and Fed-batch refers to SSF in bioreactor/fermenter.

4. LLE-OL = Liquid-liquid extraction followed by overliming

5. The maximal theoretical yield was assumed by Wyman et al. [136] to be only 95% due to cell growth.

**Table 3:** Brief summary of SSF experiments carried out on both hexose and pentose sugars.

**Raw material**	**Type of pretreatment**	**Amount of solids**	**Detoxification**	**Temp (°C)**	**Strain**	**Cultivation on hydrolyzate**	**Final ethanol conc. (g L^-1^)**	**Ethanol yield (%) ^1^**	**Mode of operation ^2^**	**Year and reference**
Barley straw	Steam	7.5% WIS	No	35	*S. cerevisiae *TMB3400	Yes	22.0	63	Batch	2007 [155]
Wheat straw	Steam	7% WIS	No	34	*S. cerevisiae *TMB3400	Yes	32.9	75	Batch	2008 [113]
Wheat straw	Steam	7% WIS	No	34	*S. cerevisiae *TMB3400	Yes	34.7	78	Fed-batch	2008 [113]
Wheat straw	Steam	9% WIS	No	34	*S. cerevisiae *TMB3400	Yes	33.2	59	Batch	2008 [113]
Wheat straw	Steam	9% WIS	No	34	*S. cerevisiae *TMB3400	Yes	38.1	71	Fed-batch	2008 [113]
Sugar cane bagasse	Steam	7.5% WIS	No	32	*S. cerevisiae *TMB3400	Yes	26.7	59 *	Batch	2008 [128]
Sugar cane bagasse	Steam	7.5% WIS	No	35	*P. stipitis *CBS6054	Yes	19.5	43 *	Batch	2008 [128]
Corn stover	Steam	10% WIS	No	35	*S. cerevisiae *TMB3400	Yes	30.3	54	Batch	2006 [112]
Corn stover	Steam	11% WIS	No	35	*S. cerevisiae *TMB3400	Yes	36.8	59	Fed-batch	2006 [112]

1. Based on maximal theoretical ethanol yield on available hexoses and pentoses (in most cases glucose and xylose).

2. Batch and fed-batch refers to SSF in bioreactor/fermenter.

### Substrate loading

**Figure 3:**
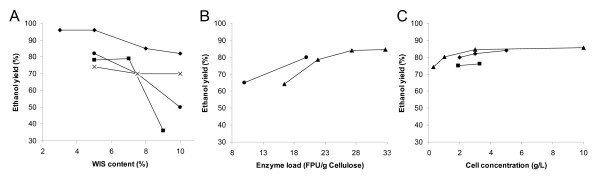
The influence of substrate loading (A), enzyme loading (B), and cell concentration (C) on ethanol yield in SSF of different materials: pretreated barley straw (black circle) [121], pretreated spruce (black diamond) [117, 149], pretreated salix (black square) [120], pretreated willow (black triangle) [122] and pretreated corn stover (×) [150].

In order to achieve a high final ethanol concentration, a high substrate loading, and hence a high WIS content, is crucial for the economy of the SSF process. Batch mode is the classical form of SSF. When the WIS content in SSF is increased, the ethanol yield tends to decrease (Figure 3A). In practice, it has been difficult to achieve good ethanol yields above WIS contents of around 10% (cf. Tables 2 and 3).

Instead of adding all substrate initially, a gradual or stepwise addition, *i.e*. a fed-batch approach can be used. There are several advantages by running SSF in fed-batch mode. By not adding all the hydrolyzate at once, the levels of inhibitors can be kept lower, giving less inhibition of the fermentation. A suitable feed rate may also allow a continuous conversion of inhibitors, as has been shown in fed-batch fermentation of dilute-acid hydrolyzates [114]. In addition, it has been reported that also the inhibition of the enzymes decreases when some of the toxic compounds are converted [51]. Stirring is a significant problem at high WIS contents due to the high viscosity [115], which results in mass and heat transfer problems. This becomes less pronounced with fed-batch SSF, due to the gradual hydrolysis of added fibers [116,117]. An additional advantage with fed-batch, is that the glucose level can be kept lower during co-fermentation of xylose and glucose (SSCF), which promotes xylose uptake [112,113] (as discussed later on). An alternative to fed-batch addition is to make a pre-hydrolysis, *i.e*. to add enzymes to the bioreactor some time before the fermenting organism is added. This can be made at an elevated temperature and will decrease the initial viscosity at the start of fermentation [118]. A disadvantage may be a less efficient co-fermentation of xylose due to the higher glucose concentration in the medium in the case of SSCF.

### Enzyme loading

The enzyme loading is clearly important for the process economy, but the economic sensitivity towards the enzyme loading in SSF is difficult to predict due to the large uncertainties of the cost of enzymes, and lack of sufficient experimental data on the effect of enzyme load. Techno-economical calculations have indicated that a 50% reduction of enzyme loading is beneficial if the yield decreases less than 6–7% and required residence time is not increased by more than 30% [119]. The enzymatic hydrolysis of the solid fraction has a large control over the total rate of ethanol production in SSF [117,120]. Studies in which the enzyme loading has been varied therefore show a strong positive correlation between enzyme loading and the overall ethanol yield [121,122] (Figure 3B). Commercial cellulase preparations available today often need to be supplemented with extra β-glucosidase to prevent end-product inhibition by cellobiose. Optimal β-glucosidase additions have been estimated for *e.g*. saccharification of pretreated aspen [123]. The optimal enzyme cocktail composition is certainly raw-material specific, and supplementation with extra β-glucosidase is – as to be expected – more important in SHF than in SSF [124].

To decrease the amount of added enzymes needed, investigations of SSF with mixtures of *S. cerevisiae *and the β-glucosidase producing yeast strain *Brettanomyces clausenii*, have been undertaken, and compared to SSF with only *S. cerevisiae*. At low enzyme loadings and without β-glucosidase addition, the mixture performed well. However, at higher cellulase loadings, higher ethanol yields were obtained when β-glucosidase was added [125]. Another way of overcoming limiting hydrolysis and simplify the SSF process, is to use cellobiose-fermenting yeasts, such as *Brettanomyces clausenii *[126], or possibly recombinant *Klebsiella oxytoca *[127].

### Yeast loading

In a large-scale SSF process, the yeast (or other fermenting microorganisms) will most likely be cultivated on the hemicellulose hydrolyzate (see Figure 1). Hence, a higher yeast concentration in the SSF will result in a lower overall ethanol yield if the substrate cost for the production of the yeast is considered. However, lowering the yeast concentration will lower the volumetric productivity, and may also lead to a stuck fermentation. The rate of the enzymatic hydrolysis have in many – probably most – reported SSF experiments been rate determining, and the yeast concentration could therefore be lowered [117,119,120]. In agreement with this, there seems not to be a strong positive correlation between cell concentration and measured ethanol yield (not counting the yield cost of the yeast production or sugar losses in the pre-treatment) above 1–2 g L^-1 ^cells (Figure 3C) for typical SSF conditions (~10% WIS and 30 FPU g^-1 ^cellulose). There is no doubt more work to be done on balancing the rates of hydrolysis and fermentation during SSF.

### Co-fermentation of pentose and hexose sugars (SSCF)

Progress is rapid in the field of xylose fermentation, but few industrial yeast strains have yet the demonstrated capability of fermenting xylose in lignocellulosic hydrolyzates efficiently. Hahn-Hägerdal *et al*. [92] recently presented information on the performance of industrial xylose fermenting strains in lignocellulosic hydrolyzates. All strains covered in their summary were XR and XDH expressing strains, which also overexpressed XK. TMB3400 is the only industrial pentose fermenting *S. cerevisiae *strain for which results on SSF of lignocellulosic materials have so far been reported [112,113,128]. Ethanol concentrations reaching 40 g L^-1 ^and yields up to 80% of the theoretical based on xylose and glucose (at a WIS content of 7%) have been achieved (Table 3). By-product formation decreases the ethanol yield from xylose with xylose fermenting strains of *S. cerevisiae*. However, less xylitol is formed by XR/XDH-carrying strains in fermentation of lignocellulosic hydrolyzates [129,130] compared to defined medium, probably due to additional electron acceptors present in the media. This was seen also in SSF experiments with the strain TMB3400 for several xylose-rich materials [112,113,128]. Both glycerol and xylitol formation lead to a regeneration of NAD^+ ^(cf. Figure 2). Interestingly, more glycerol than xylitol was produced [113].

Other pentose utilizing yeasts than *S. cerevisiae *TMB3400 have been evaluated in SSCF. Recently, Rudolf et al. [128] used sugar cane bagasse as a substrate in SSF with *P. stipitis *as a fermenting organism (see Table 3). It was indeed possible to use the organism in untreated bagasse hydrolyzate, but clearly higher yields and ethanol concentrations were achieved with *S. cerevisiae *TMB3400. Xylose fermenting bacteria have not been much examined in lignocellulosic SSF, but yellow poplar hardwood was used in SSF experiments with recombinant *Z. mobilis *co-fermenting xylose and glucose [131]. However, a thorough detoxification was required prior to the SSF.

Arabinose fermentation in SSF has not yet been reported, although arabinose fermenting *S. cerevisiae *strains have recently been constructed [132,133] as well as strains co-utilizing arabinose and xylose [134]. Also *Z. mobilis *strains co-utilizing arabinose and xylose have been developed [135]. However, further work is needed before efficient ethanol production in SSF from arabinose can be conducted.

### Temperature

In SSF a compromise between the optimal temperatures for the cellulolytic enzymes and the yeast is needed. Earlier SSF experiments in our labs were often run at a temperature of 37°C. Since the yeast *S. cerevisiae *has an optimal temperature around 30°C and the cellulolytic enzymes around 55°C, this was regarded as a suitable compromise at the high end of what *S. cerevisiae *can tolerate [117,120,122,125,136]. However, recent studies have shown important strain differences with respect to temperature tolerance, and furthermore, the co-fermentation of glucose and xylose is affected by temperature. Rudolf *et al*. [128] concluded that more xylose was consumed by TMB3400 at 32°C than at 37°C during SSF of sugar cane bagasse, and Olofsson *et al*. [113] found that a temperature of 34°C was to prefer in SSF of wheat straw. Possibly, a lower rate of hydrolysis, which gives a slower release of glucose, favors xylose uptake in the competition for transporters. Furthermore, inhibition effects may play a role, and tolerance to inhibitors may be higher at temperatures closer to the optimum of the yeast.

Thermotolerance is clearly an important topic for SSF and thermotolerant yeast strains, *e.g. Fabospora fragilis, Saccharomyces uvarum, Candida brassicae, C. lusitaniae, and Kluyveromyces marxianus*, have been evaluated for future use in SSF [137-141], to allow fermentation at temperatures closer to the optimal temperature for the enzymes. However, in all these cases saccharification of pure cellulose (*e.g*. Sigmacell-50) or washed fibers, in defined fermentation medium, were applied. SSF of cellulose with mixed cultures of different thermotolerant yeast strains have also been carried out [140,142]. However, there is so far a lack of results from SSF experiments in which untreated lignocellulosic materials have been used together with thermotolerant strains.

### Inhibitors

The amounts and types of inhibitory compounds vary strongly between different raw materials, and also depend on the pretreatment method. Hence, the needed inhibitor tolerance of a strain in an SSF process may vary depending on raw material. Several alternatives of detoxification (*i.e*. removal of inhibitory compounds) have been explored, *e.g*. over-liming, extraction with organic solvents, ion exchange, molecular sieves, and steam stripping [143,144]. Overliming with Ca(OH)_2 _is the most commonly used method. A significant drawback of this method is that calcium salts may precipitate in the process and contaminate surfaces of distillation columns, evaporators and heat-exchangers. Hence, detoxification should be avoided if possible, due to additional process cost as well as possible loss of fermentable sugars [145,146].

More tolerant yeast strains for SSF than those available today, may be achieved through genetic modifications, *e.g*. overexpressing genes encoding enzymes for resistance against specific inhibitors, and altering co-factor balance in the cell [7]. Another way to improve strains is by evolutionary engineering, through which strain robustness is improved by mutation and selection [147]. Yet another approach to overcome the problem with inhibition is by adapting the SSF process. By applying *e.g*. a fed-batch mode of substrate addition with proper feed protocol and control variables, the levels of inhibitors can be kept at an acceptable level. Such strategies have proven successful during cultivation and fermentation of liquid hydrolyzates [148,149], as well as in SSF [117]. A combination of more inhibitor-tolerant strains in combination with efficient feed strategies will likely improve process robustness in SSF processes.

## Conclusion

**Figure 4:**
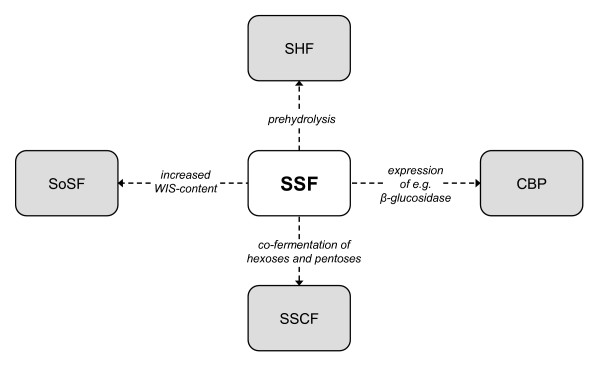
SSF in relation to other process options. The arrows show the approach of SSF to other process options as a result of process changes. (Abbreviations: SSF = simultaneous saccharification and fermentation; SHF = separate hydrolysis and fermentation; CBP = consolidated bioprocessing, *i.e*. a process in which the enzymes are produced by the fermenting organism; SSCF = simultaneous saccharification and co-fermentation; SoSF = solid state fermentation.)

The basic challenges for SSF – as for any other process option – are to obtain as high degree of hydrolysis and as high ethanol yield as possible. There is no doubt that the development of more efficient pentose fermenting yeasts with improved robustness in hydrolyzates, and the development of more efficient enzymes and enzyme cocktails will continue. Process economic evaluations are essential for this development. Useful "iso-cost" curves in the operational space can thereby be constructed to guide further development work [119]. The simplest – and original – SSF is a batch process in which substrate, enzymes and yeast are all present in the reactor initially, and at the intended concentrations. Additional degrees of freedom are available for process improvement by changing some of the initial conditions. In principle, substrate(s), enzymes and even yeast may all be *gradually *fed during the process. Several of these options can probably be discarded for practical reasons, but it is nevertheless clear that there are many options relatively unexplored for the improvement of SSF. The new variants of SSF that are now tried, can be seen as a move of the "classical" SSF process in the direction of other process options, although not taking it all the way (see Figure 4). The result will be new "hybrid" processes, which will be tuned for the feedstock and the enzymes used.

## Competing interests

GL has research grants from the Swedish Energy Agency for experimental investigations on SSF processes and also participates in EU financed projects on this topic. GL is co-author of one patent concerning improved inhibitor tolerance of *S. cerevisiae*.

## Authors' contributions

KO and MB contributed equally to the writing. KO wrote the parts concerning fermentation and pentose fermentation, whereas MB wrote the parts concerning pretreatment and enzymatic hydrolysis. GL set the scope and outline of the paper, wrote the introduction and conclusion, and revised the manuscript. All authors read and approved the final version.
